# Comparing the thermal stability of 10-carboxy-, 10-methyl-, and 10-catechyl-pyranocyanidin-3-glucosides and their precursor, cyanidin-3-glucoside

**DOI:** 10.1038/s41538-022-00131-9

**Published:** 2022-02-18

**Authors:** Danielle M. Voss, Gonzalo Miyagusuku-Cruzado, M. Mónica Giusti

**Affiliations:** Department of Food Science and Technology, The Ohio State University, 2015 Fyffe Road, Columbus, OH 43210-1007 USA

**Keywords:** Chemistry, Natural products

## Abstract

Pyranoanthocyanins are vibrant, naturally derived pigments formed by the reaction of an anthocyanin with a cofactor containing a partially negatively charged carbon. This study compared the thermal stability and degradation products of 10-carboxy-pyranocyanidin-3-glucoside (pyruvic acid cofactor), 10-methyl-pyranocyanidin-3-glucoside (acetone cofactor), and 10-catechyl-pyranocyanidin-3-glucoside (caffeic acid cofactor) with their anthocyanin precursor to evaluate the role of the pyranoanthocyanin C_10_ substitution on stability. Pyranoanthocyanins exhibited absorbance half-lives ~2.1–8.6 times greater than cyanidin-3-glucoside, with ~15–52% of their original pigment remaining after 12 h of 90 °C heating at pH 3.0. 10-Methyl-pyranocyanidin-3-glucoside was the most stable (*p* < 0.01) based on UHPLC-PDA analysis, while 10-catechyl-pyranocyanidin-3-glucoside had the most stable color in part due to contribution from a colored degradation compound. Protocatechuic acid formed in all heated samples, which suggested a similar degradation mechanism among pigments. In conclusion, the C_10_ substitution impacted the extent of pyranoanthocyanin stability and the degradation compounds formed.

## Introduction

Pyranoanthocyanins (PACNs) are anthocyanin (ACN)-derived pigments characterized by an additional pyran ring located between the ACN C_4_ and C_5_ –OH group. First identified in aged red wine^1,2^, PACNs play an important role in wine’s color stability and the development of a tawny hue during aging^3^. In addition to wine, PACNs have been found in aged juice^4–6^, sumac^7^, and strawberries^8^. PACNs produce vibrant colors across pH values^9^ and have reported antioxidant and anti-inflammatory properties^10^.

**Fig. 1:**
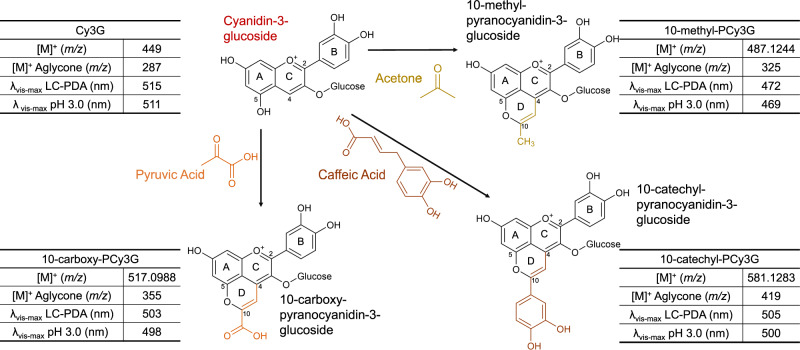
Chemical structure of the pigments evaluated with the cofactors used in the formation of pyranoanthocyanins. Pigment characteristics were determined at time 0 from UHPLC-PDA-ESI-MS/MS and QTOF-MS analysis (for PACN *m*/*z*) and spectrophotometric analysis (at pH 3.0 adjusted with HCl). Cy3G cyanidin-3-glucoside, 10-carboxy-PCy3G 10-carboxy-pyranocyanidin-3-glucoside, 10-methyl-PCy3G 10-methyl-pyranocyanidin-3-glucoside, 10-catechyl-PCy3G 10-catechyl-pyranocyanidin-3-glucoside.

PACNs are formed by a reaction between an ACN with a free –OH group on C_5_ and a reactive cofactor with an enolizable or vinyl group^11^. Compounds that may serve as cofactors for PACN formation include acetone, a solvent used for pigment extraction^12^; pyruvic acid, a fermentation metabolite^13^; and caffeic acid, a naturally occurring hydroxycinnamic acid^14,15^. The chemical structures of the formed PACNs with these cofactors are in Fig. 1. To initiate PACN formation, cycloaddition occurs between the partial negative carbon on the reactive cofactor and the positive charge at C_4_ of the ACN, occurring through resonance-based charge delocalization^11^. By adjusting the reaction conditions, PACN formation efficiency can increase. The solution pH, incubation temperature, cofactor concentrations, cofactor types, and presence and strain of yeast can influence PACN yields^16–20^. Under accelerated formation conditions, defined by higher incubation temperatures and cofactor concentrations and pH at 3.1, caffeic acid was the most efficient cofactor for formation^21^, yielding 10-catechyl-PACNs.

PACNs show promise as naturally derived colorants due to their improved stability over precursor ACNs. This improved stability includes color retention across pH values^9^ and resistance to bleaching by ascorbic acid^22^. PACNs also have improved stability to heat. Methyl-pyranoanthocyanidins retained ~55% absorbance after heating for 1 h at 80 °C, whereas their corresponding anthocyanidins completely degraded^23^. Similarly, malvidin-derived PACNs had degradation half-lives ~3.5–7 times greater than the parent anthocyanin after 98 °C heating in pH 1 aqueous solutions^9^. Research has focused on malvidin-derived PACNs due to their abundance in wine^11^; however, cyanidin-3-glucoside (Cy3G) is the most abundant ACN in nature^24^ making it a readily available reactant for PACN formation.

During heating, anthocyanins in the flavylium cation conformation undergo C_2_ hydration and transition into the colorless chalcone^25^, the same transition that occurs with pH increases^26,27^. The unstable chalcone is then cleaved into degradation compounds with phloroglucinaldehyde forming from the A-ring and protocatechuic acid forming from the B-ring, as is the case with cyanidin-derived ACNs^28–30^. Knowledge on PACN’s thermal degradation pathways and degradation compounds is limited, however. In studies focusing on the pH structural transitions of PACNs, only a few have reported data consistent with a C_2_-hydrated PACN^31–33^ with most current studies not detecting a hydrated PACN following pH jumps^34–37^.

With the limited evidence for the occurrence of the hydration initiated, chalcone-mediated degradation pathway in PACNs, Cy3G derived PACNs were hypothesized to have improved thermal stability and form different degradation compounds than Cy3G. The objective of this experiment was to evaluate thermal stability using multiple analysis methods and to identify the degradation compounds of three PACNs (10-carboxy-, 10-methyl-, 10-catechyl-pyranoCy3G) in comparison to their anthocyanin precursor, Cy3G, to explore the role of the C_10_ substitution on PACN stability. This work will help to expand understanding of PACN’s degradation mechanism and will contribute to the understanding of how chemical structure can influence their behavior, important information for PACN’s continued development as colorants.

## Results and discussion

### Pigment identification and purity determination

PACNs were successfully formed from the reaction between Cy3G (from elderberry) and pyruvic acid, acetone, or caffeic acid (Fig. 1), although the formation conditions and efficiency differed greatly depending on the reactants. The identities of the formed PACNs were assigned by a combination of the following information: the relative retention time, the wavelength of maximum absorption in the visible light range (*λ*_vis-max_), accurate mass-per-charge (*m*/*z*) value, MS/MS fragmentation patterns, and comparison to reported literature. The formed PACNs had later retention times than Cy3G under the reverse-phase ultra-high-performance liquid chromatographic (UHPLC) gradient used; this is consistent with a decrease in polarity by the addition of the second pyran ring and C_10_ substitution. The *λ*_vis-max_ of the PACNs was hypsochromically shifted compared to Cy3G (Fig. 1) which is consistent with previous work on PACNs^21^. The greatest shift observed was for 10-methyl-pyranoCy3G (10-methyl-PCy3G) in which the *λ*_vis-max_ was 43 nm shorter than Cy3G. The *m*/*z* for each PACN was greater than Cy3G, consistent with the addition of the second pyran ring. To increase confidence in pigment identification, the accurate mass per charge values were obtained by quadrupole time of flight (QTOF) mass spectrometry for the PACNs. The *m*/*z* of 10-carboxy-pyranoCy3G (10-carboxy-PCy3G; 517.0988) was ~68 *m*/*z* units higher than Cy3G, corresponding to the additional pyran ring and carboxy substitution. The *m*/*z* of 10-methyl-PCy3G (487.1244) was ~38 *m/z* units higher than Cy3G, consistent with the addition of the second pyran ring and methyl substitution. The *m*/*z* of 10-catechyl-pyranoCy3G (10-catechyl-PCy3G; 581.1283) was the highest with a *m/z* ~132 units greater than Cy3G, consistent with the addition of a pyran ring and catechyl substitution. For all pigments, MS/MS analysis showed a fragment with a *m*/*z* 162 units lower (287, 355, 325, and 419 for Cy3G, 10-carboxy-PCy3G, 10-methyl-PCy3G, and 10-catechyl-PCy3G, respectively), consistent with the loss of glucose to form the aglycone. The identified *m*/*z* values and MS/MS fragmentation patterns are consistent with the values reported in Blanco-Vega et al. for these cyanidin-derived PACNs^38^. All isolates had purity >96% based on the 260–700 nm max plot chromatograms.

### Pigment stability challenges during sample preparation and room temperature storage

Cy3G showed great solubility and stability during sample preparation and at room temperature, but the PACNs presented unique challenges related to their specific chemical structures. PACNs have previously been reported to precipitate following extended periods of room temperature storage in aqueous solutions^34,39^. In the present study, the observed precipitation and color changes were initially reverted by including 1.5% acidified (0.01% HCl) MeOH in solutions with pH 3.0 water. Isolated 10-carboxy-PCy3G precipitated during preparation, and therefore, it was solubilized and stored in acidified MeOH. After mixing with pH 3.0 water and after 12 h at room temperature, 10-carboxy-PCy3G experienced a 23.6 ± 12.4% drop in absorbance at *λ*_vis-max_, a 29.1 ± 16.2% apparent reduction in the total pigment content (determined spectrophotometrically), and a total color change (Δ*E*_Lab_) of 11.5 ± 7.1 driven by a decrease in chroma with a Δ*E*_Lab_ of 5 considered a discernable color change^40^. Interestingly, no degradation compounds were observed in the UHPLC-PDA chromatogram. These results suggested 10-carboxy-PCy3G precipitated from the aqueous solution rather than degrading, but visual observation and confirmation of precipitation was obscured by the amber microcentrifuge tubes used for storage to minimize light degradation. At pH 3.0, 10-carboxy-PACNs are reported as zwitterionic^41^; this loss in repulsive charges on the 10-carboxy-PACNs may have led to aggregation and precipitation after 12 h of storage^37^.

Brown coloration was observed upon thawing the 10-catechyl-PCy3G concentrated isolate. By mixing the concentrate in acidified MeOH prior to pH 3.0 water dilution, the color was reverted to its characteristic orange color. Yet, after standing at room temperature in an aqueous solution, the brown color reappeared with a Δ*E*_Lab_ of 7.0 ± 4.5 after 6 h and 12.5 ± 6.0 after 12 h. This color change was accompanied by an increase in absorbance near ~550–700 nm (Supplementary Figure 1) and a decrease in absorbance at the *λ*_vis-max_ by 14.5 ± 8.0% and 25.3 ± 9.4% after 6 and 12 h, respectively. Surprisingly, these changes were neither reflected by a significant decrease in pigment content (*p* = 0.74) nor by the presence of new peaks on the UHPLC-PDA chromatograms. Based on previous reports, it was hypothesized that these PACNs aggregated through non-covalent interactions at room temperature^37^. The acidification and dilution associated with both pigment content and HPLC analyses may have reverted this aggregation.

In contrast, 10-methyl-PCy3G was stable following freezing and thawing and at room temperature with a ΔE_Lab_ of just 1.5 ± 0.4 and no new peaks detected in the UHPLC-PDA chromatogram after 12 h. The pyran ring C_10_ substitution largely impacted room temperature stability. The changes observed with 10-carboxy- and 10-catechyl-PCy3G appeared to be reversible, allowing for the pigments to be recovered. Consequentially, additional processing steps may be needed for use of these PACNs at room temperature.

### Pigment thermal stability by spectrophotometric measurements

#### Color expression

**Fig. 2:**
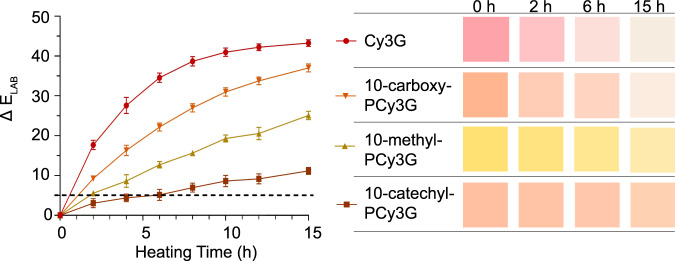
Δ*E* across 15 h of 90 °C heating compared to time 0. Results are expressed as means (*n* = 3) ± standard deviation. Color swatches represent pigment color at pH 3.0 based on L*a*b* color characteristics across heating with data presented in Table 1.

Each of the four pigments produced distinct colors when mixed in pH 3.0 water (acidified with HCl and with 1.5% acidified MeOH) with initial color parameters provided in Table 1 and represented in the color swatches in Fig. 2. For Cy3G, the initial color was characterized by a vibrant red-orange color (hue angle (*h*_ab_°) = 18.0 ± 0.2°). In contrast, 10-methyl-PCy3G was yellow (*h*_ab_° = 90.7 ± 0.1°). The 10-catechyl-PACN and 10-carboxy-PACN produced similar orange colors (*h*_ab_° = 46.5 ± 0.5° and *h*_ab_° = 48.9 ± 0.2°, respectively), but the chroma value (*c*_ab_*) for 10-carboxy-PCy3G was 6.8 units higher than 10-catechyl-PCy3G indicating a more saturated color.

**Table 1:** Color characteristics from CIE-L*c_ab_*h_ab_* color system for pigments across 15 h of 90 °C heating.

Pigment	Heating time (h)	L*	*c*_ab_*	*h*_ab_°
Cy3G	0	77.8 ± 0.3^a^	42.8 ± 0.7^a^	18.0 ± 0.2^a^
	2	84.7 ± 0.2^b^	26.5 ± 0.4^b^	18.6 ± 0.3^a^
	6	91.1 ± 0.2^c^	11.8 ± 0.5^c^	37.1 ± 1.8^b^
	15	94.4 ± 0.1^d^	7.5 ± 0.2^d^	81.2 ± 1.3^c^
10-carboxy-PCy3G	0	82.3 ± 0.2^a^	43.6 ± 1.0^a^	48.9 ± 0.2^a^
	2	84.8 ± 0.1^b^	34.8 ± 0.5^b^	47.1 ± 0.2^a^
	6	88.7 ± 0.2^c^	22.4 ± 0.3^c^	47.2 ± 0.4^a^
	15	94.0 ± 0.1^d^	8.8 ± 0.1^d^	62.5 ± 1.5^b^
10-methyl-PCy3G	0	90.1 ± 0.1^a^	57.3 ± 1.0^a^	90.7 ± 0.1^a^
	2	91.1 ± 0.2^b^	52.0 ± 1.3^b^	91.9 ± 0.2^b^
	6	91.7 ± 0.1^c^	44.8 ± 0.6^c^	92.7 ± 0.1^c^
	15	92.8 ± 0.1^d^	32.5 ± 1.0^d^	93.7 ± 0.1^d^
10-catechyl-PCy3G	0	83.6 ± 0.5^a^	36.8 ± 1.2^a^	46.5 ± 0.5^a^
	2	85.0 ± 0.2^b^	34.3 ± 0.6^ab^	46.0 ± 0.1^a^
	6	85.9 ± 0.4^b^	32.5 ± 1.1^b^	48.6 ± 0.3^b^
	15	88.1 ± 0.2^c^	27.8 ± 0.6^c^	55.2 ± 0.5^c^

Different letters show statistical differences between time points within one pigment (*p* < 0.05).

With increasing heating time, the color of the samples became lighter (increasing L* values), increased in hue angle, and decreased in chroma (Table 1). For Cy3G, 10-carboxy-PCy3G, and 10-methyl-PCy3G, a Δ*E*_Lab_ of 5 was reached by 2 h of heating. It took 6 h of 90 °C heating for a discernible color change with 10-catechyl-PCy3G (Fig. 2).

#### Full-spectrum absorbance

**Fig. 3:**
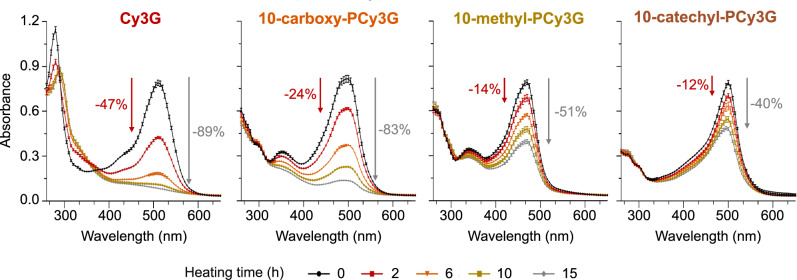
Full spectrum absorbance (260–700 nm, 5 nm increments) for isolated pigments heated at 90 °C in pH 3.0 solution with data points representing means (*n* = 3) ± standard deviation. Percentage values represent a reduction in absorption at *λ*_vis-max_ after 2 h (in red) and 15 h (in gray) compared with 0 h. For abbreviations, see Fig. 1.

**Fig. 4:**
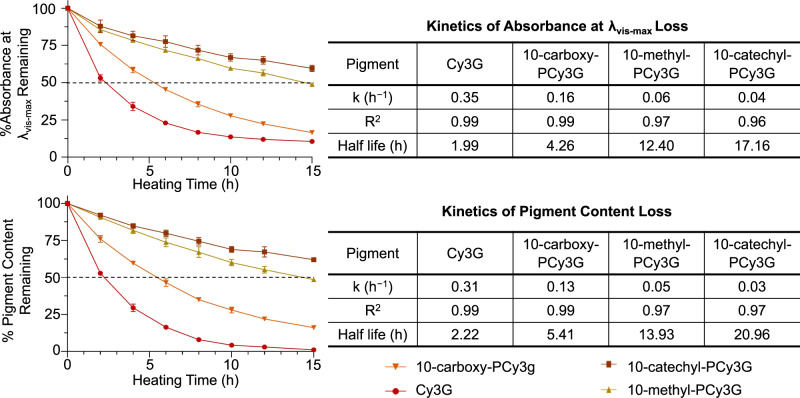
Change in absorbance at *λ*_vis-max_ and pigment content across 90 °C heating at pH 3.0. Data points represent mean (*n* = 3) ± standard deviation. Kinetic parameters modeled absorbance and pigment content change following first-order kinetics. For abbreviations, see Fig. 1.

Significant changes were observed in Cy3G’s absorbance spectra (260–700 nm) across 15 h of 90 °C heating (Fig. 3). Absorbance decreased in the range of ~260–285 nm and in the visible light region, likely due to the loss of flavylium cation due to its conversion into colorless equilibrium forms and degradation compounds^42^. At the *λ*_vis-max_, Cy3G absorbance was reduced by ~47% after only 2 h of heating (Fig. 3), consistent with Cy3G’s thermal instability observed in previous studies^43,44^. After 10 h of heating, degradation rates appeared to plateau with only an additional ~3% change in absorption over the subsequent 5 h of heating (Fig. 4). First-order kinetics were used to model Cy3G’s decrease in absorbance at the *λ*_vis-max_ with a strong coefficient of determination for the regression fit (*R*^2^ = 0.99), consistent with the understanding that ACN degradation follows first-order kinetics under high-temperature heating^28,43,45,46^. The calculated half-life (1.99 h) is similar to that reported by Sadilova et al. (1.82 h) who modeled Cy3G thermal degradation under similar conditions (pH 3.5, 95 °C) with HPLC-DAD-MS/MS^29^. The slight differences may be due to the lower pH (3.0) and temperature used in the present study (90 °C).

In contrast, the absorption of Cy3G increased in the ~290–365 nm region during the 15 h of heating. The increased absorption in the 320–340 nm range may be attributed to the formation of the colorless chalcone^25,42^, a proposed intermediary in thermal degradation^47^. In addition, the increased absorption observed at 290 nm may be related to the formation of ACN degradation compounds that have an absorption maxima in this region^25^.

For the three PACNs across the heating time, a decrease in absorption was observed from ~325 to 700 nm (Fig. 3) with the magnitude of change significantly lower for PACNs than Cy3G at all time points (*p* < 0.01). The biggest change occurred in the *λ*_vis-max_ region where after 2 h, absorbances were reduced by 24.4 ± 1.0%, 14.4 ± 2.0%, and 12.1 ± 4.1% for 10-carboxy-PCy3G, 10-methyl-PCy3G, and 10-catechyl-PCy3G, respectively. These reductions were ~2–3.9 times less than the reduction observed for Cy3G after 2 h heating. Degradation continued with heating time, following first-order kinetic parameters; after 15 h of heating, absorbance at the *λ*_vis-max_ was reduced by 83.5 ± 0.6%, 51.2 ± 0.1%, and 40.4 ± 2.1% for 10-carboxy-PCy3G, 10-methyl-PCy3G, and 10-catechyl-PCy3G, respectively, with significant differences among all pigments (*p* < 0.01; Figs. 3 and 4). PACN absorbance loss at *λ*_vis-max_ with heating was modeled with first-order kinetics, consistent with previous work on PACN thermal degradation^9,48,49^. In the present experiment, the PACNs did not reach their degradation plateau by 15 h. Therefore, the kinetic model used was constrained to the plateau value obtained from the regression of Cy3G degradation (absorbance at *λ*_vis-max_ = 0.086) to better predict the end point of complete pigment loss. There were slight changes in absorbance in the UV region for PACNs; however, the changes were less than those observed for Cy3G and limited information has been reported on UV-absorption’s relation to PACN structural changes.

#### Pigment content

In contrast to the full spectrum absorption, pigment content represents the sample’s absorbance at pH 1 at their respective *λ*_vis-max_. Despite the differences in methodology between these spectrophotometric techniques, similar results were obtained showing the superior stability of PACNs. After 2 h of heating, 10-catechyl-PCy3G and 10-methyl-PCy3G were reduced by less than 10% in comparison to a ~24% reduction for 10-carboxy-PCy3G and ~47% reduction for Cy3G (Fig. 4). After 15 h of 90 °C heating, Cy3G pigment content was reduced by nearly 100%, though some pigment remained in the PACN samples with 10-catechyl-PCy3G having the smallest change across heating (*p* < 0.01). As with absorbance at *λ*_vis-max_, pigment content loss was modeled with first-order kinetics for both Cy3G and the PACNs, with strong coefficients of determination for each fit (*R*^2^ ≥ 0.97), and the kinetic model used for the PACNs was constrained to the plateau reached by Cy3G of 0.119. This finding is consistent with previous work that has modeled ACN pigment content following first-order kinetics^50–52^. The calculated half-life for Cy3G based on pigment content analysis (2.22 h) is shorter than the one calculated in Cao et al. (3.04 h) following 90 °C degradation of purified Cy3G in pH 3.5 buffer^43^. The differences may be due to the influence of buffer on the thermal stability as well as differences in heating conditions and lengths between these two experiments.

### Pigment stability based on high-performance liquid chromatography (HPLC) and photodiode array detection

**Fig. 5:**
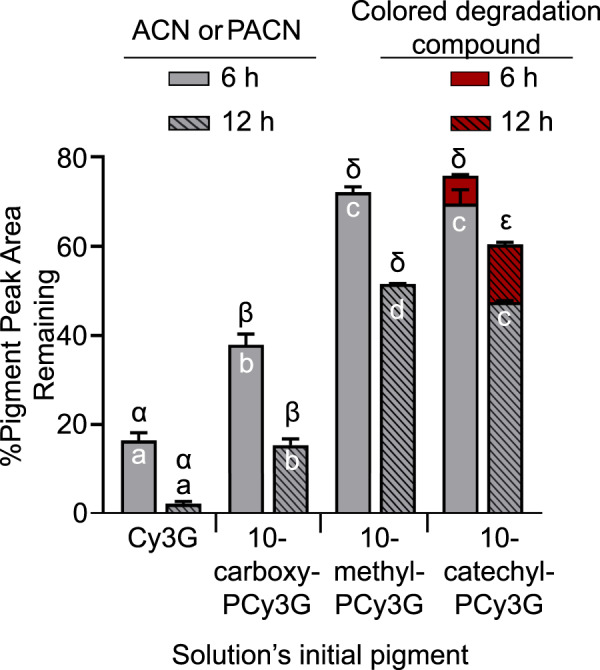
Change in pigment peak area based on UHPLC-PDA 260–700 nm max plot chromatogram after 6 and 12 h 90 °C heating at pH 3.0. Bars represent mean (*n* = 3) ± standard deviation. Latin letters represent statistical differences at one time point excluding the pigmented degradation compounds; Greek letters represent statistical differences at one time point when including the peak area of the colored degradation compounds. For abbreviations, see Fig. 1.

Colored degradation compounds have previously been reported to form from PACN degradation both with oxidative degradation^53^ and ascorbic acid bleaching^22^. To account for the potential influence of colored degradation compounds on the spectrophotometric stability, the change in peak area across heating time for Cy3G and each PACN was compared. After 6 h of heating, 10-methyl-PCy3G and 10-catechyl-PCy3G were more resistant to degradation than 10-carboxy-PCy3G and Cy3G (*p* < 0.01; Fig. 5). After 12 h of heating, 10-methyl-PCy3G had the smallest change in peak area, statistically smaller than with 10-catechyl-PCy3G (*p* < 0.01; Fig. 5), suggesting that it was most resistant to degradation. Looking at the total area of pigmented compounds, however, 10-catechyl-PCy3G had statistically more pigment remaining after 12 h of heating due to contribution from a colored degradation compound (*λ*_max_ = 478 nm). The color produced by this degradation compound likely contributed to the enhanced stability of 10-catechyl-PCy3G observed in the spectrophotometric measurements.

Regardless of the criteria used, PACNs showed superior heat stability with calculated absorbance and pigment content half-lives ~2.1–9.4 times larger than Cy3G, consistent with the conclusions of the previous studies^9,23,39,53^. PACN’s improved thermal stability may be related to the decreased likelihood for ring-opening hydration to occur. This process, one of the first steps leading to ACN degradation^47^, has been previously reported to be minimized in PACNs due to decreased electrophilicity of C_2_ occurring as a result of the increased positive charge delocalization into the additional pyran ring^34^. As a result, PACNs may have remained for a longer time in the flavylium cation configuration, continuing to produce color during heating.

The superior stability of 10-catechyl-PCy3G and 10-methyl-PCy3G compared to 10-carboxy-PCy3G may be related to the influence of C_10_ substitutions on C_2_ electrophilicity. The catechol moiety may increase positive charge delocalization more so than a methyl or carboxy substitution^54^. A methyl substitution is an electron-donating group^55^ whereas a carboxy substitution withdraws electrons^41^. These effects alter the electron density of C_2_ and maybe influence the rate of hydration and PACN thermal degradation. The least stable PACN evaluated was 10-carboxy-PCy3G, formed with the pyruvic acid cofactor. Despite the lower observed stability, carboxy PACNs are some of the most studied PACNs due to their formation and abundance in aged red wine^56,57^.

Sun et al. reported that 10-methyl-pyranomalvidin-3-glucoside was more stable than 10-catechyl-pyranomalvidin-3-glucoside and 10-carboxy-pyranomalvidin-3-glucoside across 5 h of 98 °C heating in pH 1.0 aqueous solution^9^. In the present work, performed at pH 3.0 and 90 °C, there were no significant differences between 10-catechyl- and 10-methyl-PCy3G’s absorbance stability after 6 h of heating (*p* = 0.057). Significant differences between these two compounds began at hour 8 (*p* = 0.047) with 10-catechyl-PCy3G more stable than 10-methyl-PCy3G based on spectrophotometric measurements. The additional heating time used in the present experiment and the different pH values may explain these discrepancies in reported stability.

### Degradation compound identification

#### Degradation compounds from cyanidin-3-glucoside

**Fig. 6:**
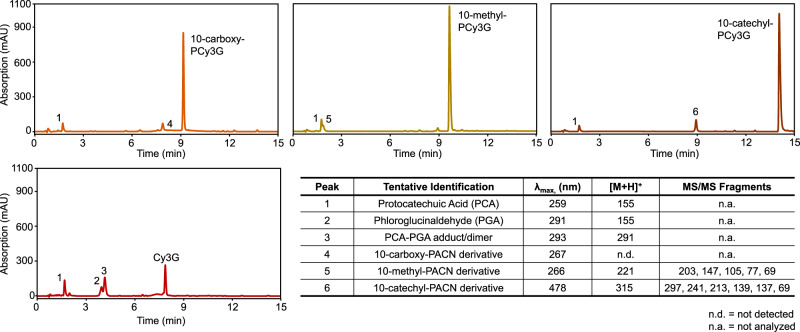
UHPLC-PDA chromatograms of pigment isolates following 6 h of 90 °C heating. Injection volume for Cy3G was half of the injection volume used for the three pyranoanthocyanins. Information in the table was obtained from UHPLC-PDA-ESI-MS/MS analysis.

Cy3G at pH 3.0 is known to degrade with heat into an A-ring degradation compound (phloroglucinaldehyde) and a B-ring degradation compound (protocatechuic acid) following a pathway involving C_2_ hydration of the flavylium cation and breakdown of the formed chalcone intermediary^28,29,58^. In the present study, protocatechuic acid (Peak 1; *λ*_max_ of 259 nm; *m*/*z* of 155) and phloroglucinaldehyde (Peak 2; *λ*_max_ of 291 nm; *m*/*z* of 155) were identified to have formed during heating of Cy3G using UHPLC-PDA-ESI-MS/MS analysis and confirmation with analytical standards (Fig. 6). While a chalcone or chalcone glycoside was not identified during our UHPLC-PDA-ESI-MS/MS analyses, its formation was suggested in our full spectrum measurements denoted by an increase in the 320–340 nm range^25,42^. A third degradation compound, Peak 3, was tentatively identified as either a dimer of protocatechuic acid or a dimer of phloroglucinaldehyde or an adduct between the two compounds with a *λ*_max_ of 293 nm and *m*/*z* of 291, consistent with the combination of protocatechuic acid and/or phloroglucinaldehyde and the corresponding loss of 1 water molecule (−18 *m*/*z*). This adduct has previously been proposed following thermal heating of cyanidin-derived ACNs under similar heating conditions (pH 3.5, 95 °C)^29,30^. ACNs have also been observed to degrade through quinoidal base degradation into a coumarin product^59,60^, though no similar compounds were identified in the present study.

#### Pyranoanthocyanin degradation compounds

Less is known on PACN’s thermal breakdown pathway and degradation compounds than those of ACNs. He et al. identified syringic acid and several colored degradation compounds forming with 6 months of room temperature storage at pH 3.6 of malvidin-derived PACN-flavanols and identified oxovitisin possibly from oxygen-induced degradation of 10-carboxy-pyranomalvidin-3-glucoside^53^. However, to our knowledge, no additional identification of thermal degradation compounds of PACNs has been reported. In addition, many previous studies have not identified a hydrated PACN with pH jumps^34–37^. Therefore, a different degradation pathway than that observed with ACNs was initially hypothesized to occur with PACNs. Interestingly, in the present experiment, protocatechuic acid (Peak 1; *λ*_max_ of 259 nm; *m*/*z* of 155), was identified in all three heated PACN samples (Fig. 6). As this was the same degradation compound identified in heated Cy3G, a similar degradation pathway involving C_2_ hydration and chalcone breakdown is suggested to occur for PACNs with heating. While hydration may be less likely to occur in PACNs due to the extended charge delocalization^34^, we hypothesize that the additional energy provided to the system by 90 °C heat was enough to overcome the activation energy barrier for hydration to occur with heating. Hydration is a reported endothermic process in ACNs^61^, supporting our hypothesis that energy would be required to initiate the hydration process with PACNs. As with Cy3G, an intermediary chalcone PACN was not identified in the present study.

In addition to protocatechuic acid, an additional degradation compound, unique to each PACN, was identified to form with heating (Fig. 6). In heated 10-carboxy-PCy3G, a degradation compound with *λ*_max_ of 267 nm and an earlier retention time (~7.8 min) was detected (Peak 4). The *m*/*z* of this unknown compound was not detected; however, targeted analysis for a *m*/*z* of 251 showed that the *m*/*z* was not consistent with the *m*/*z* differences between PACN types (64 *m*/*z* difference between a 10-carboxy-PACN and 10-catechyl-PACN, 30 *m*/*z* difference between 10-carboxy-PACN and 10-methyl-PACN). Future work should focus on the additional characterization of this compound. The unique degradation compound that formed in heated 10-methyl-PCy3G (Peak 5) had a *λ*_max_ of 266 nm with a secondary, shorter peak at 349 nm and a *m*/*z* of 221. MS/MS fragmentation of Peak 5, named as a 10-methyl-PACN derivative, yielded major fragment ions of 203 and 147, a loss of 18 *m*/*z* units and 74 *m*/*z* units, respectively, from the base peak, and 105, 77, and 69. In heated 10-catechyl-PCy3G, the unique degradation compound (Peak 6) had a *λ*_max_ of 478 nm and *m*/*z* of 315 and was labeled as a 10-catechyl-PACN derivative in Fig. 6. MS/MS fragmentation of 10-catechyl-PACN derivative showed major fragment ions of 297, 241, 213, 139, 137, and 69 with the *m*/*z* of the first two fragment ions listed corresponding to a loss of 18 *m*/*z* units and 74 *m*/*z* units, respectively, from the base peak. Additionally, this compound was not identified in a neutral loss scan for a glucose moiety (162 *m*/*z*), suggesting glucose was cleaved during a prior degradation step. The 10-methyl-PACN derivative and 10-catechyl-PACN derivative share similar MS/MS fragmentation patterns and the *m*/*z* difference between the two compounds is 92 *m*/*z* units, the same known difference between 10-catechyl- and 10-methyl-PACNs. These consistencies in MS/MS fragmentation patterns and *m*/*z* values suggested that these two compounds may form from the same degradation pathway which is hypothesized to include C_2_ hydration and the formation of a PACN chalcone intermediary. The degradation compound is believed to contain parts of the PACN A and D-ring and the attachment at C_10_. We proposed that the C_10_ substitution influenced the rate of C_2_ hydration and chalcone degradation but did not change the pathway. The formation of a colored degradation compound, the 10-catechyl-PACN derivative, is significant as it likely contributed to the improved color, absorbance, and pigment content stability observed with 10-catechyl-PCy3G. This compound may help to explain the discrepancy in stability patterns when comparing spectrophotometric data to UHPLC-PDA peak area changes. Electron conjugation between multiple rings, as could be contributed by the catechol substitution, could produce the color observed by this compound.

In conclusion, all three PACNs evaluated showed exceptional thermal stability with pigment and color remaining after 15 h of 90 °C heating. These results are in contrast to Cy3G, which nearly completely degraded under these heating conditions. The PACN C_10_ substitutions impacted the extent of stability and the formed degradation compounds. 10-Methyl-PCy3G, formed with acetone cofactor, exhibited the greatest stability based on UHPLC-PDA analysis and 10-catechyl-PCy3G, formed with caffeic acid, showed the greatest color stability based on spectrophotometric readings. A colored degradation compound formed only in 10-catechyl-PCy3G contributed to the improved stability observed in spectrophotometric readings. Protocatechuic acid was identified as a degradation compound in both ACN and PACN samples suggesting that PACNs degraded following a similar pathway to ACNs involving hydration. These results showed that the chemical structure of the PACN, determined by the cofactor used for formation, affected the thermal stability which may have implications for their continued development as naturally derived colorants and their application in foods. Consequentially, future studies will focus on how the chemical structure of the PACN B-ring, determined by the anthocyanin used for formation, impacts thermal stability.

## Methods

### Materials

Commercial elderberry ACN powder (*Sambucus nigra*) was provided by D. D. Williamson (Louisville, KY, USA). Acetone was obtained from Fisher Scientific (Waltham, MA, USA). Caffeic acid and syringic acid were obtained from Sigma-Aldrich (St. Louis, MO, USA). Protocatechuic acid and phloroglucinaldehyde were obtained from Aldrich Chemistry (Milwaukee, WI, USA). 4-Hydroxybenzoic acid was obtained from Acros Organics (Fair Lawn, NJ, USA). All reagents used were of analytical grade or higher unless otherwise noted.

### Pyranoanthocyanin formation

Elderberry was selected as the ACN source for PACN formation due to its high content of Cy3G^17^. An ACN extract was prepared by dissolving the ACN powder in acidified water. The 10-carboxy-PACNs were formed following the procedure detailed in Hoehn with slight modifications^16^. The ACN extract and pyruvic acid were mixed at a 1:200 (ACN: cofactor) molar ratio, and the pH of the final solution was adjusted to 3.1, previously reported as optimal for the formation of PACNs^16^. The solution was incubated at room temperature for 15 days. 10-Methyl-PACNs were formed following the procedure in Sun et al. with modifications^9^. The ACN extract was mixed with acetone at a 1:50 (ACN: cofactor) molar ratio and the pH was adjusted to ~3.1. The solution was incubated at room temperature for ~45 days. 10-Catechyl-PACNs were formed following the procedure in Straathof and Giusti modified by Miyagusuku-Cruzado et al.^17,21^. The ACN extract was mixed with caffeic acid at a 1:30 (ACN: cofactor) molar ratio in pH 3.1 acidified water. The solution was incubated at 45 °C for up to 4 days.

### Compound semi-purification and isolation

After PACN formation, each solution was semi-purified using solid-phase extraction on Waters Sep-pak C18 cartridges (Milford, MA, USA) following the procedure in Rodriguez-Saona and Wrolstad with no ethyl acetate rinse^62^. Solvent was evaporated from semi-purified extracts using a Büchi Rotavapor (New Castle, DE, USA) at 40–50 °C and/or a Vacufuge Plus vacuum evaporator at 30 °C (Eppendorf, Hamburg, Germany).

The target compound in each semi-purified solution was isolated using a Prominence semi-preparative HPLC system comprised of two LC-6AD pumps, a CBM-20A controller, a SIL-20A HT autosampler, a CT0-20A column oven, and an SPD-M20A PDA detector (Shimadzu, Columbia, MD, USA). Chromatographic separation was achieved using a binary mobile phase system composed of A: 4.5% aqueous formic acid and B: acetonitrile at a flow rate of 12 mL/min. Either a Synergi Max-RP 80 Å 4 μm, 250 × 21.2 mm column or Luna 5 μm PFP 100 Å, 250 × 21.20 mm column (both from Phenomenex, Torrance, CA, USA) was used for separation with different elution gradients. Following isolation, samples were concentrated following the solid phase extraction procedure described previously. Cy3G, 10-catechyl-PCy3G, and 10-methyl-PCy3G were frozen as aqueous concentrates. Isolated 10-carboxy-PCy3G was observed to precipitate. Therefore, the solution was centrifuged and decanted, and the pellet was dissolved in acidified MeOH (0.01% HCl) and stored in a freezer.

### Heat treatments

The isolated ACN and PACN concentrates were thawed by sonication. Cy3G, 10-catechyl-PCy3G, and 10-methyl-PCy3G were mixed with acidified MeOH and all four pigment solutions were diluted in pH 3.0 water (acidified with HCl) to a final MeOH concentration of 1.5%. Each was then adjusted to a pH of 3.00 ± 0.05 with the addition of 1 M NaOH and 2 N HCl and, after sufficient time for pH equilibration, to an absorbance of 0.82 ± 0.05 at each respective *λ*_vis-max_ listed in Fig. 1. Equal aliquots were transferred into amber microcentrifuge tubes and flushed with nitrogen to minimize light and oxygen-induced degradation.

Heating was conducted in a thermostatic water bath (Fisher Scientific, Waltham, MA, USA) with water temperature measured at 90.0 ± 2.5 °C throughout. Microcentrifuge tubes were removed every 2 h up to 12 h with the final sample removed after 15 h. Tubes were chilled in ice for 5 min and equilibrated to room temperature for 5 min prior to measurements. Controls were prepared in a similar manner but stood at room temperature for 6 and 12 h prior to measurements.

### Spectral and pigment content readings

Spectral readings were conducted using a SpectraMax M2 plate reader (Molecular Devices, San Jose, CA, USA). Samples were read in Nunc™ 96-well UV Transparent Microplates (Thermo Scientific, Waltham, MA, USA) from 260 to 700 nm with 5-nm increments and from 465 to 515 nm with 1-nm increments for a targeted reading of the region containing the *λ*_vis-max_. Color data, expressed in the CIE-L*c_ab_*h_ab_* color system, was calculated using the ColorBySpectra^63^ software under D65 illuminant and 10° observer angle from the 380–700 nm absorbance reading. Color swatches were produced using the Adobe Color software (Adobe, San Jose, CA, USA) based on the L*a*b* values calculated by ColorBySpectra^63^. Total color change (Δ*E*_Lab_) was calculated using the CIE-L*a*b* values.

Total pigment content was measured using the pH differential method described in Giusti and Wrolstad with PACN measurements conducted only at pH 1.0^64^. Values were calculated as mg Cy3G equivalents/L using the molar absorptivity (26,900) and molecular weight (449.2 g/mol) of Cy3G for all pigments^64^.

For stability analysis, the percentage of changes in absorption at *λ*_vis-max_, pigment content values, and pigment peak areas were compared and calculated using the following formula where *A*_ti_ is the value after heating and *A*_t0_ is the initial value.

1 $$\frac{{A_{\rm{ti}}}}{{A_{{\rm{t}}0}}} \ast 100 = \% \;{\rm{Change}}$$

### UHPLC and mass spectrometric analysis

Compound identification, purity determination, and pigment and degradation compound monitoring were conducted using a Nexera-i LC2040C 3D ultra HPLC with photodiode array detection coupled to a LCMS-8040 triple quadrupole mass spectrometer with electrospray ionization (UHPLC-PDA-ESI-MS/MS) (Shimadzu, Columbia, MD, USA). Chromatographic separation was achieved using an Ultra IBD column 1.9 μm, 50 × 2.1 mm (Restek, Bellefonte, PA, USA) with UltraGuard C18 guard cartridge 10 × 2.1 mm (Restek, Bellefonte, PA, USA) and a column oven set to 50 °C. Mobile phases comprised of 4.5% formic acid in water (A) and acetonitrile (B) at a rate of 0.25 mL/min. Purity was determined using the following gradient for chromatographic separation: 0–30% B from 0.01 to 15 min, 30–45% B from 15 to 20 min, 45–45% B from 20 to 23 min followed by 5 min of column equilibration. Pigment purity was based on the peak areas from the 260 to 700 nm max plot chromatogram^18^. Monitoring of compounds during heating and degradation compound identification was achieved under the following chromatographic separation conditions: 0–20% B from 0.01 to 12 min, 20–35% B from 12 to 15 min, 35–45% B from 15 to 16 min, 45% B from 16 to 19 min followed by an additional 4 min of column equilibration. For comparison of peak areas, the 260–700 nm max plot chromatogram was used at all time points and Eq. 1 was used for calculations.

Mass spectrometry analyses were performed under the following parameters: 200 °C heat block, 230 °C desolvation line, 1.5 L/min nebulizing gas flow, and 15 L/min drying gas flow. Each run used a total ion scan under positive ion mode and negative ion mode from 100–1200 *m*/*z* (negative mode was only used for compound identification), a precursor ion scan in positive mode with –35 eV collision energy for the pigment aglycones (listed in Fig. 1), and selective ion monitoring under positive mode for the pigments and expected degradation compounds. For unreported degradation compounds from PACNs, product ion scans with collision energies from –10 to –50 eV and a neutral loss scan for glucose loss (*m*/*z* of 162) were used. Lab Solutions software (Shimadzu, Columbia, MD, USA) was used for the analysis of PDA and mass spectrometric results.

Accurate mass values were provided by the Nutrient & Phytochemical Analytical Shared Resource of The Ohio State University Comprehensive Cancer Center (NPASR). Isolated samples of the three PACNs were analyzed with an Agilent Infinity 1290 UHPLC with diode array detector tandem to an Agilent 6550 QTOF mass spectrometer (Santa Clara, CA, USA). Chromatographic separation was achieved using a CSH C18 column 1.8 μm, 150 mm × 2.1 mm (Waters, Milford, MA, USA) and a column oven at 40 °C. Mobile phases comprised of 5% formic acid in water (A) and 5% formic acid in acetonitrile (B) at a rate of 0.3 mL/min under the following gradient: 0% B from 0–1.5 min, 0–60% B from 1.5–15 min, 60–95% B from 15–16 min. followed by 4 min of column equilibration. QTOF-MS analyses were performed under the following parameters: 150 °C drying gas, 18 L/min drying gas flow, 350 °C sheath gas, 12 L/min sheath gas flow, 1000 V nozzle, and 3500 V capillary. The exact mass was acquired from 30 to 1700 amu at ~ 20,000 resolution and 3 Hz. Within run calibration of *m*/*z* 1033 from HP921 and *m*/*z* 112 from trifluoroacetic acid were used. MassHunter Acq software version B.09 was used for acquisition and to centroid profile mass data and MassHunter Qual ver. B.10 as used for data analysis (both from Agilent, Santa Clara, CA, USA).

### Statistical analysis

A one-way analysis of variance with Bonferroni multiple comparison post hoc test was used for comparisons between pigments and time points with nested analyses used when possible. Absorbance and pigment content value decrease with heating was modeled using first-order kinetic parameters. Experiments were conducted in triplicate with results presented as mean ± standard deviation throughout. Analyses were performed using Graph Pad Prism (GraphPad Software, San Diego, CA, USA) and a *p* value < 0.05 was considered significant.

## Supplementary information

Supplemental Material

## Data Availability

The datasets generated during and/or analyzed during the present study are available from the corresponding author on reasonable request.
