# Supplementary material for: Comparing the thermal stability of 10-carboxy-, 10-methyl-, and 10-catechyl-pyranocyanidin-3-glucosides and their precursor, cyanidin-3-glucoside
Source: NPJ Sci Food. 2022 Feb 18;6:16. doi: 10.1038/s41538-022-00131-9 (PMC8857255; doi:10.1038/s41538-022-00131-9)
Supplement: Supplementary file 1 — Supplemental Material [file 41538_2022_131_MOESM1_ESM.pdf]

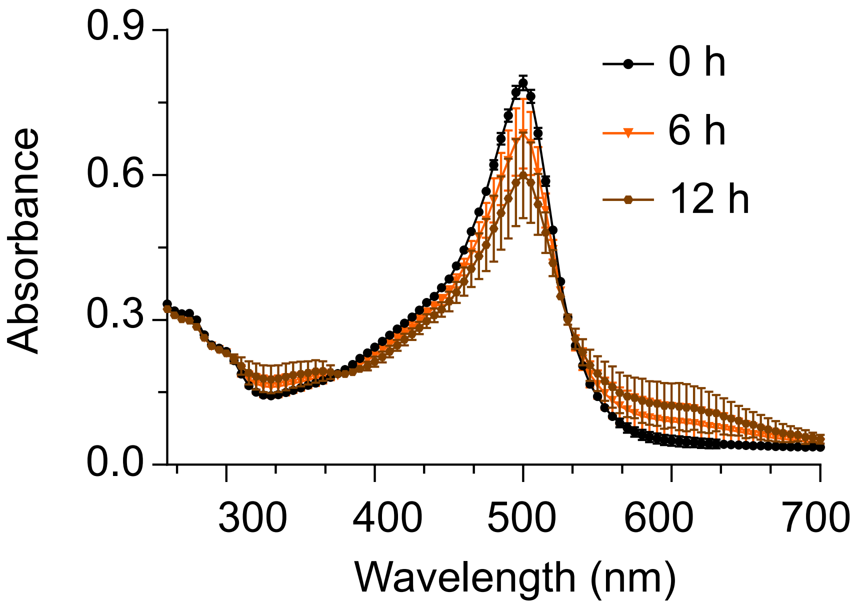

Supplementary Figure 1: Full spectra absorbance (260–700 nm, 5 nm increment) for isolated 10-catechyl-pyranocyanidin-3-glucoside at pH 3.0 for up to 12 h at room temperature. Data points represent means (n = 3) ± standard deviation.
